# 

**Published:** 1891-01-17

**Authors:** 


PRICE ONE PENNY.

o. 225, Vol. IX.—January 17th, 1891.

[Registered at the General Post Office as a Newspaper for transmission in the United Kingdom and Abroad.]

WITH EXTRA NURSING SUPPLEMENT.

Per Annum, 6s. 6d.; post free.

Monthly Parts, 6d.; post free 8d.

Ditto per Annum, 8s., post free.

### THE HOSPITAL

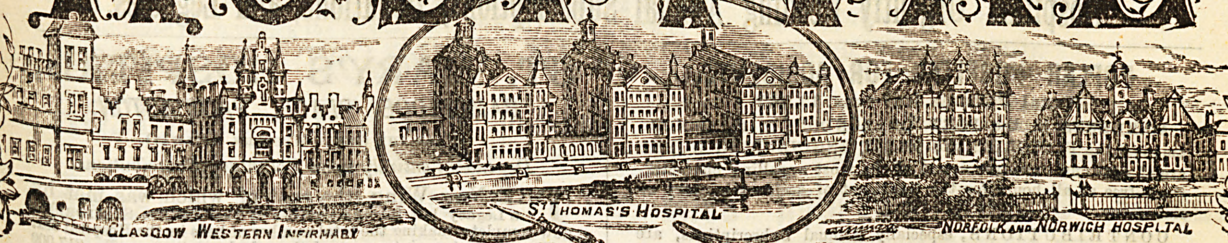

A WEEKLY JOURNAL OF

### Science, Medicine, Nursing and Philanthropy.

### SATURDAY, JANUARY 17th, 1891.

**Suggestions to the Lords** - - - - - - - 243
**Passing Topics.**—The New Loves gain the Day - - - 244
**The Doctor's Armchair.**—Winter Feedings - - - 245
**The History and Capabilities of Herbal Simples.** By W. T. Fernie, M.D.—XXVII. The Common Daisy - - 246
**Criminals: How to Make and How to Mend Them** - 247
**The Story of the Insane from Year to Year** - - 248
**Books Received** - - - - - - - - - 248
**Everybody's Page** - - - - - - - - 249
**Notes and News** - - - - - - - - - 250
**Annotations.**—Philip Henry Gosse—Medicos under the Microscope—A Children's Ailing Home at Aberdeen - - 252
**The Practitioner's Mirror and Retrospect:—**
Medicine.—The Approach of Cholera, 253; Erysipelas: New Methods of Treatment, 254; Cold Treatment of Pneumonia - - - - - - - - - - 255
Surgery.—Tubercular Diseases of the Bones and Joints - 255
Therapeutics.—Ouabaïne - - - - - - - 256
The Practitioner's Bookshelf.—Grubor on Ear Diseases 257
**Scraps and Gleanings** - - - - - - - - 257
**The Student of Medicine.**—Editorial Notes—Leading Athletes—Notes from the Schools—Appointments—Students' Medical Societies—Presentation to a Medical Student—The Students' Bookshelf, &c. - - - - - 258

### EXTRA SUPPLEMENT—THE NURSING MIRROR.

**En Passant.**—Ashton-under-Lyne—A Privileged Profession—Belfast Nurses' Home—Short Items—A Queensland School—Glasgow goes Forward - - - - lxxxiii

**Lectures on Surgical Ward Work and Nursing—** By Alex. Miles, M.B.Edin., C.M., F.R.C.S.E.—Lecture X.—A Spray Dressing - - - - - - - - lxxxiv

**Death in our Ranks** - - - - - - - - lxxxiv

**Notes from Australia** - - - - - - - lxxxv

**Appointments** - - - - - - - - - lxxxv

**Everybody's Opinion.**—Christmas Fare in Workhouses—Asylum Attendants—Ladies' Committees - - - lxxxvi

**For Reading to the Sick.**—Encouragement - - lxxxvi

**Christmas Parcels—Presentations** - - - - lxxxvii

**Keeping Christmas** - - - - - - - - lxxxvii

**The Nurses' Bookshelf.**—A Guide to Massage—The Care of the Sick - - - - - - - - lxxxviii

**Notes and Queries** - - - - - - - - lxxxviii

**Amusements and Relaxations** - - - - - lxxxviii

